# Black Pepper or Radish Seed Oils in a New Combination of Essential Oils Modulated Broiler Chickens’ Performance and Expression of Digestive Enzymes, Lipogenesis, Immunity, and Autophagy-Related Genes

**DOI:** 10.3390/vetsci9020043

**Published:** 2022-01-24

**Authors:** Asmaa T. Y. Kishawy, Hanan S. Al-Khalaifah, Hend S. Nada, Elshimaa M. Roushdy, Asmaa W. Zaglool, Tamer Ahmed Ismail, Seham M. Ibrahim, Doaa Ibrahim

**Affiliations:** 1 Department of Nutrition and Clinical Nutrition, Faculty of Veterinary Medicine, Zagazig University, Zagazig 44511, Egypt; seham.mohamed@zu.edu.eg; 2 Environment and Life Sciences Research Center, Kuwait Institute for Scientific Research, P.O. Box 24885, Safat 13109, Kuwait; hkhalifa@kisr.edu.kw; 3 Departments of Microbiology, Faculty of Veterinary Medicine, Zagazig University, Zagazig 44519, Egypt; hend.saeed@hotmail.com; 4 Department of Animal Wealth Development, Animal Breeding and Production, Faculty of Veterinary Medicine, Zagazig University, Zagazig 44511, Egypt; emroshdy@zu.edu.eg; 5 Department of Animal Wealth Development, Genetic and Genetic Engineering, Faculty of Veterinary Medicine, Zagazig University, Zagazig 44511, Egypt; asmaa.wagih2008@gmail.com; 6 Department of Clinical Laboratory Sciences, Turabah University College, Taif University, P.O. Box 11099, Taif 21944, Saudi Arabia; t.ismail@tu.edu.sa

**Keywords:** essential oils, black pepper, radish seed oil, digestibility, lipogenesis, autophagy, chickens

## Abstract

Optimal combinations of essential oils (EOs) can enhance performance and maintain poultry productivity. The effects of EOs with black pepper oil (BPO) or radish seed oil (RSO) on performance and the expression of digestive enzymes, lipogenesis, immunity, and autophagy-related genes in broiler chickens were explored. Six dietary treatments for 300 one-day-old chicks were allocated as follows: controls were fed a basal diet, one group was fed an EO-supplemented diet (1.5 g/kg diet of parsley, mint, and carrot seed oils (1:1:1)), and other groups received Eos + BPO0.25, Eos + BPO0.5, Eos + RSO0.25, and Eos + RSO0.5 treatments, with a basal diet containing EOs plus BPO or RSO at the level of 0.25 or 0.5 g/kg, respectively. Supplementation with 0.5 g/kg of EOs plus BPO or RSO resulted in the most improved maximum BWG and FCR in broiler chickens. The lactobacilli population was increased in Eos + BPO0.5, followed by EOs + RSO0.5, unlike in the control. The highest expression of the *CCK* and *PNLIP* genes was identified in the Eos + BPO group. The *FAS* and *ACC* genes were upregulated, while the *IgA* and *IL-10* genes were downregulated, with EOs plus RSO or BPO. The group that received Eos + BPO0.5, followed by Eos + RSO0.5, displayed patterns of higher expression for *atg5*, *atg7*, and *atg12*, with lower expression of mTOR. In summary, a new combination of EOs with 0.5 g/kg BPO had potential growth-promoting and immune-boosting effects in broiler chickens.

## 1. Introduction

Recently, phytogenic plants have shown promising perspectives for modulating immune responses, digestive-enzyme activity, gut signaling molecules, and the microbiota composition, consequently increasing poultry productivity and health [1,2]. Aromatic plants, herbs, spices, and extracts including their essential oils (EOs) have been used in animal production as alternatives to antibiotics. Examples of plant-derived natural bioactive compounds include EOs that are volatile oils or easily evaporated benzene or terpene derivatives that are normally obtained from plant material by steam or water distillation [3]. Previous studies investigated the roles of many EOs combined in poultry feed as growth promoters and digestive stimulants [4], antimicrobial agents [5], antioxidants [6], antiparasitic agents [7], antioxygenic agents [8], hypocholesterolemic agents [9], and immunostimulants [10]. However, using a new blend comprising mint, parsley, carrot seed, black pepper, and radish seed oils has not been investigated until now. 

Black pepper (*Piper nigrum*) is a common spice added to food to increase palatability and digestibility [11]. The most potent compounds of black pepper are cupsisin, cupsaeesin, cupsantine, and piperine [11]; it is also rich in glucose-6-phosphate dehydrogenase and glutathione peroxidase, which exert antioxidant and immunostimulant effects in broiler chickens [12]. Piperine has anti-ache [13] and digestive-stimulant effects [14], and it also increases the absorption of selenium, vitamin B complex, β-carotene, and other nutrients [15]. Additionally, supplementation with piperine upregulated the expression of IL-10 and IgA, thus decreasing the pathogenic bacterial load and inflammation in broiler chickens, in accordance with [16]. Dietary supplementation with black pepper enhanced the performance, nutrient digestibility, feed conversion ratio, and carcass-meat safety and quality in poultry [17]. From our point of view, piperine has antimicrobial, anti-inflammatory, and antioxidant activities.

Radish seeds (*Raphanus Sativus*) contain alkaloids such as coumarins, saponins, flavonoids, and anthocyanins [18]. These alkaloids reduce uric acid levels in the blood, which is correlated with circulating markers of inflammation and free-radical reactions [19]. The anthocyanins are an essential group of nutritional antioxidants that have many physiological functions, as they defend living cells from oxidative damage and subsequently help to prevent diseases [20]. Radish seeds also contain isothiocyanates that have antimicrobial, antimutagenic, and antiatherosclerotic activity [21]. Additionally, carrot seeds contain a number of active ingredients such as tannins, steroids, flavonoids, terpinene, pinene, myrcene, carotene, and carotol [22] that have antioxidant and immunostimulant effects [23]. Parsley leaves contain high amounts of active ingredients such as vitamin C, vitamin A, B-complex vitamins, iron salts, calcium, iodine, apigenin, flavonoids, and myristicin, which is the common active component of parsley oil [24]. Myristicin has been proved to have antioxidant, antibacterial, and immunomodulatory effects, as well as acting as a digestive stimulant, as it improves the liver’s secretion of bile [24,25,26,27]. Additionally, phytogenic blends containing parsley improved broiler chickens’ growth performance [28]. The analysis of peppermint leaves showed that they contain volatile oils (approximately 0.5–4.0%), comprising 50–78% free menthol, monoterpene, thymol, and menthofurane [29], which have antibacterial [30], fungicidal, digestion-stimulating, and appetizing properties [31,32]. 

From this perspective, in the current study, we aimed to investigate different mechanisms that regulated the potential impact of the previously mentioned EOs on broiler chickens’ performance, especially at the molecular level. A gap in the knowledge between the use of dietary EOs and the expression of digestive-enzyme-related genes exists. Moreover, their role in controlling autophagy and related genes inside the bird’s body needs to be examined. Therefore, the aim of the present study was to explore the effect of an EO blend of carrot seed, parsley, and peppermint oils with the addition of black pepper oil or radish seed oil as a potential feed additive in broiler chickens’ diets. From this perspective, its effects on growth performance, digestibility, and immunity and its mode of action in regulating the expression of digestive enzymes, fatty acids, immunity, and autophagy-related genes were investigated.

## 2. Materials and Methods

### 2.1. Ethical Approval

Birds care and management and experimental measures were in conformity with guidelines and ethics of the Institutional Animal Care and Use Committee (ZU-IACUC2/f/76/2021) of the Faculty of Veterinary Medicine at Zagazig University.

### 2.2. Birds’ Management, Diets, and Experimental Design

A total of 600 one-day-old chicks of a commercial meat type (ROSS-308) were obtained from a local hatchery, Sharkia Province, Zgazig, Egypt. On arrival, they were weighed and randomly allocated to six equally sized treatment groups. Each group contained 5 replicates, with 20 chicks in each. The birds were reared in an environmentally controlled house with sawdust as litter and at a density of 10 birds/m^2^. Continuous lighting was provided throughout the experiment. The starting temperature was 33 °C; then, the temperature was increased to 21 °C at the 3rd week, according to the Aviagen guidelines [33]. The basal diets were formulated according to the requirements stipulated in the Ross-Broiler-Pocket Guide [33]. The diets were divided into three stages (starter (1–10 days), grower (11–24 days), and finisher (25–35 days)) (Table 1). The proximate chemical analysis for the used feedstuffs and the experimental diets (moisture, crude protein, and ether extract) were carried out according to [34].

The six experimental groups were fed as follows: the NC (negative control) group was fed a basal diet without any additives; the EOs group was fed a basal diet supplemented with 1.5 g/kg diet of an EO blend with equal amounts (1:1:1) of parsley, peppermint, and carrot seed oils; the EOs + BPO0.25 group was fed a basal diet supplemented with EOs plus 0.25 g/kg diet of black pepper oil (BPO); the Eos + BPO0.5 group was fed a basal diet supplemented with EOs plus 0.5 g/kg diet of BPO; the Eos + RSO0.25 group was fed a basal diet supplemented with EOs plus 0.25 g/kg diet of radish seed oil (RSO); the Eos + RSO0.5 group was fed a basal diet supplemented with EOs plus 0.5 g/kg diet of RSO. The essential oils were obtained from Frontier Co-op, Aura Cacia® brands, 3021 78th St, Norway, Iowa, 52318, USA, and 100% pure EOs were obtained by the steam distillation of plant seeds.

### 2.3. Growth Performance

The body weight gain (BWG) for the whole experimental period = final live weight (g) − initial live weight (g). Feed conversion ratio (FCR) = amount of feed consumed (g)/body weight gain (g). Relative growth rate (RGR) = (final live weight − initial live weight/½ (initial live weight + final live weight)) × 100. Protein efficiency ratio (PER) = live weight gain (g)/protein consumed (g), as formerly stated by [35,36].

### 2.4. Digestibility Trail 

At 36 days of age, 0.5% titanium oxide, an indigestible marker, was added to the experimental finisher diets for 7 days. The excreta collected from each replicate pen were chemically analyzed for moisture, crude protein, and ether extract following the method of [34]. The concentration of titanium oxide in the excreta was measured as previously described [37]. 

Apparent nutrient digestibility = 100 − [100 × (indicator content (diet)/indicator content (feces) × nutrient content (feces)/nutrient content (diet)].

### 2.5. Sampling

At the end of the feeding trial, five birds per replicate were selected randomly, weighed, and used for the following procedures: 

Blood sample collection: blood samples were aseptically obtained from the bird’s wing vein and then divided into two parts—the first part was collected in a clean centrifuge tube containing an anticoagulant (EDTA) and used for measuring phagocytosis, while the second part was collected into a clean centrifuge tube without anticoagulant for the separation of serum for biochemical and immune-parameter analysis.

For molecular analysis: pancreatic, duodenal, jejunal, and splenic tissues and abdominal fat (including the fat tissues surrounding the proventriculus and gizzard and those lying against the inside abdominal wall and around the cloaca) were separated and kept in TRIzol reagent at −80 °C until further analysis by the RT–qPCR assay.

### 2.6. Cecal Bacterial Count

At the end of the feeding trial, cecal contents (one gram/sample) were collected from the slaughtered birds (5 birds/replicate) and serially diluted in 0.85% sterile saline solution [38]. An aliquot (0.1 mL) of each diluted sample was cultivated on a specific media as follows: nutrient agar plate (Oxoid, UK) at 37 °C for 2–3 days for total aerobic bacterial counts. MacConkey agar for (Oxoid, UK) was used for coliform counts. Man, Rogosa, and Sharpe agar plates (Oxoid, UK) after anaerobic incubation at 37 °C for 3 days were used for total lactobacilli. Next to incubation, the colonies of bacteria were counted in accordance with [39]. The counted bacteria were expressed as log_10_ CFU/g of cecal digesta.

### 2.7. Serum Biochemical Analysis 

The serum total protein, globulin, albumin, triglycerides (TGs), total cholesterol (TC), high-density lipoprotein cholesterol (HDL-C), low-density lipoprotein cholesterol (LDL-C), very-low-density lipoprotein cholesterol (VLDL-C), aspartate aminotransferase (AST), and alanine aminotransferase (ALT) were estimated using analytical kits (Spinreact Co., Santa Coloma, Spain).

### 2.8. Serum Immune Parameters and Assay of Phagocytosis

The lysozyme activity in the broiler serum was measured by an agarose-gel cell-lysis assay. ELISA kits obtained from Roche Diagnostics Co. (Indianapolis, Indiana, IN, USA) were used to measure the serum levels of immunoglobulin M (IgM, REF; 035071190) and immunoglobulin G (IgG, REF; 03507432).

A phagocytosis assay was performed according to [40], with some modifications. Briefly, blood samples were collected; then, a peripheral blood mononuclear cell layer was obtained, washed, resuspended in a Roswell Park Memorial Institute medium (RPMI-164), and supplemented with 15% fetal calf serum (FCS). Then, a monolayer of macrophages was obtained by seeding 5 × 10^6^ mononuclear cells in a 1 mL volume for culture on chambers with coverslips, which were stained and incubated for 1 h at 37 °C under 5% CO_2_ and 99% humidity. Non-adherent cells were removed by washing 3 times; then, after incubation for 24 h, the adherent macrophages were incubated under the same conditions with 1 mL of *Candida Albicans* (10^7^/mL of RPMI with 15% FCS) and then washed 3 times, fixed, and stained. Finally, 100 macrophages were counted to determine the % of phagocytic macrophages (number of phagocytic macrophages/total number of macrophages).

### 2.9. Gene Expression Analysis

Total RNA was isolated from pancreatic, duodenal, jejunal, splenic, and abdominal tissues using TRIzol reagent (Invitrogen Life Technologies, Carlsbad, CA, USA) according to the manufacturer’s instructions. The RNA quantity and quality were examined using a NanoDrop® ND–1000 spectrophotometer (NanoDropTechnologies; Wilmington, DE, USA). Complementary DNA (cDNA) was synthesized by the reverse transcription of total RNA using the QIAGEN Long Range Two-Step RT–PCR Kit, following the manufacturer’s guidelines. The primer sequences used are presented in Table 2 and were used for determining the expression of digestive-enzyme-related genes (pancreatic alpha 2A amylase (AMY2A), pancreatic lipase (*PNLIP*), cholecystokinin (*CCK*), and chymotrypsin-like elastase family, member 1 (CELA1)); lipogenesis-related genes (fatty acid synthase (*FAS*) and acetyl–coA carboxylase (*ACC*)); immunity-related genes (immunoglobulin A (IgA) and interleukin-10 (IL-10)); mechanistic target of rapamycin (mTOR); autophagy-related genes ((*atg5*, *atg7*, and *atg12*)). One microliter of cDNA and 12.5 μL of 2× SYBR Green qPCR mix with ROX from Bio-Rad (USA) were mixed, with the addition of 5.5 μL of RNase-free water and 0.5 μL of each forward and reverse gene-target primer. The expression of the target genes was normalized to that of glyceraldehyde-3-phosphate dehydrogenase (GAPDH). 

### 2.10. Statistical Analysis

Statistical tests were performed using the General Linear Model Procedure in SPSS version 21 for Windows (SPSS, Inc., Chicago, IL, USA). All data were confirmed to be normally distributed after transformation (ASIN). Post hoc Tukey tests were used to assess the differences between the means at the 5% probability level. Before analysis, the data of the cecal CFUs were converted to log10 CFU numbers. The fold change was calculated as follows: (B−A)/A, where the lowest value is A, and the highest value is B. The 2^−ΔΔCt^ method [41] was used for the calculation of the relative fold changes in the expression of the target genes. The data are expressed as the standard errors of the mean (SEMs). 

## 3. Results

### 3.1. Growth Performance

The effects of the EOs with BPO or RSO on broiler chickens’ performance throughout the growing period are shown in Table 3. The highest significant (*p* < 0.05) BWG was found in groups supplemented with EOs with 0.5 g/kg of BPO or RSO (increased by 6.4 and 8.5%, respectively, vs. the control). The feed conversion ratio was enhanced in all the groups fed the EOs plus BPO or RSO at both levels, followed by that in the group supplemented with the EOs, in comparison with that in the NC group. No significant differences in the feed intake and RGR among the different groups were observed. The most prominent increase in PER was detected in the group fed the EOs with 0.5 g/kg of BPO or RSO. 

### 3.2. Nutrient Digestibility 

Concerning the DM digestibility, the groups fed diets supplemented with the EOs plus BPO or RSO exhibited the highest (*p* < 0.05) DM digestibility. Moreover, the group supplemented with EOs plus 0.5 g/kg of BPO or RSO showed the highest (*p* < 0.05) CP digestibility. Additionally, the EE digestibility was significantly increased (*p* < 0.05) after supplementation with EOs plus BPO, especially at higher doses (Table 4).

### 3.3. Serum Biochemical Parameters

Data regarding the impact of the EOs plus BPO or RSO on serum biochemical parameters are shown in Table 5. The total protein, albumin, and globulin were prominently improved by the addition of the EOs plus BPO or RSO. Additionally, their highest levels were detected in the groups supplemented with the EOs plus 0.5 g/kg of BPO or RSO. The lowest concentrations of TAG, total cholesterol, LDL-C, and VLDL were identified (*p* < 0.05) in the group supplemented with the EOs plus BPO. By contrast, the HDL concentration was significantly increased (*p* < 0.05) in the groups fed the EOs plus BPO, followed by the groups fed the EOs plus RSO, compared with the NC group. The levels of ALT and AST showed no significant differences (*p* < 0.05) among all the experimental treatments.

### 3.4. Serum Immune Parameters

Supplementation with EOs plus BPO or RSO improved the tested serum immune parameters (Table 6). Moreover, the group fed the EOs plus BPO, especially at the level of 0.5 g/kg, showed the highest significant (*p* < 0.05) immune response, as evinced by the increased serum lysozyme activity, IgM, IgG, and phagocytic percentage, compared with the other experimental groups. 

### 3.5. Cecal Bacterial Count 

The population of beneficial lactobacilli significantly increased (*p* < 0.05) after dietary supplementation with the EOs plus BPO at the level of 0.5 g/kg, followed by that with 0.5 g/kg of EOs plus RSO, when compared with that for the NC group. Meanwhile, the mean cecal populations of total aerobic bacteria and coliforms were reduced upon increasing the levels of EOs with either BPO or RSO (Table 7). 

### 3.6. Expression of Digestive-Enzyme- and Lipogenesis-Related Genes

**Figure 1:**
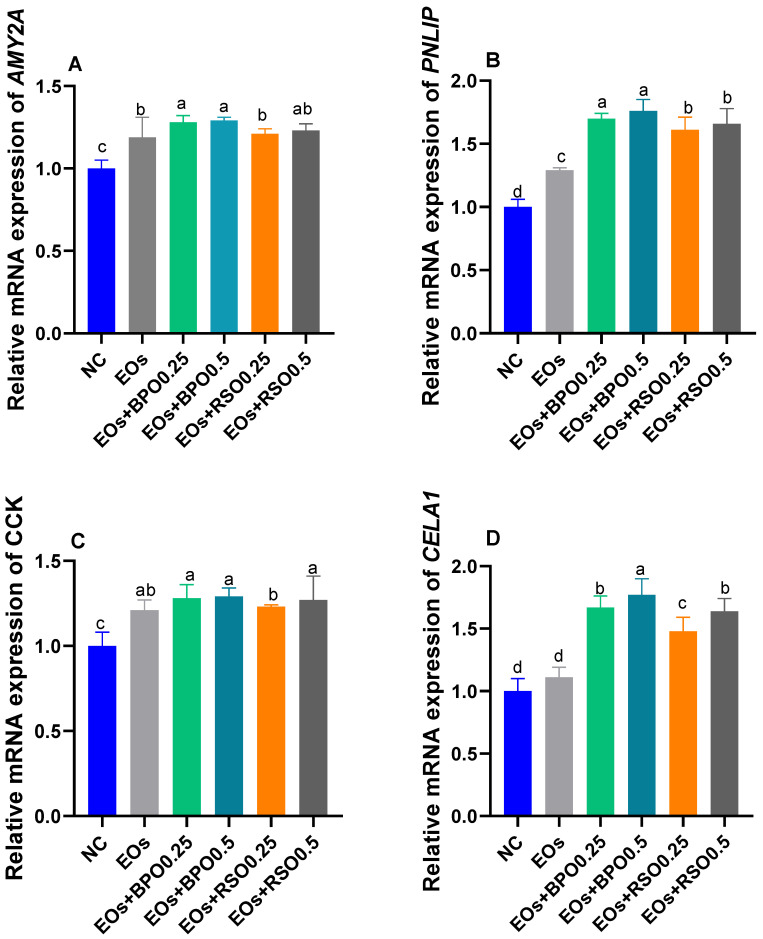
Effect of EOs with black pepper oil or radish seeds oil on mRNA expression of digestive enzymes related genes (pancreatic alpha 2A amylase (AMY2A, (**A**), pancreatic lipase (*PNLIP*, (**B**)), cholecystokinin (*CCK*, (**C**)) and chymotrypsin-like elastase family, member 1 (CELA1, (**D**)); ^abcd^ means within the same column carrying different superscripts are significantly different at (*p* < 0.05). EOs mixture of mint, parsley, and carrot oils. NC: feed basal diet without additives; EOs: birds fed a basal diet supplemented with 1.5 g/kg EOs; EOs + BPO0.25: birds fed a basal diet supplemented with 1.5 g/kg EOs plus 0.25 g/kg black pepper oil (BPO); EOs + BPO0.5: birds fed a basal diet supplemented with 1.5 g/kg EOs plus 0.25 g/kg black pepper oil (BPO); EOs + RSO0.25: birds fed a basal diet supplemented with 1.5 g/kg EOs plus 0.25 g/kg radish seed oil (RSO); EOs + RSO0.5: birds fed a basal diet supplemented with 1.5 g/kg EOs plus 0.5 g/kg radish seed oil (RSO).

Data related to the mRNA expression of digestive enzymes are presented in Figure 1. The mRNA expression of pancreatic AMY2A (a) was upregulated (*p* < 0.05) in response to the inclusion of 0.5 g/kg of RSO and both levels of BPS, compared with the NC group. The highest expression (*p* < 0.05) of the *CCK* and *PNLIP* genes was found in the groups supplemented with the EOs plus BPO (increased by 1.29 and 1.76 fold). The groups fed the EOs plus BPO or RSO showed significant upregulation (*p* < 0.05) of the mRNA expression of CELA1.

**Figure 2:**
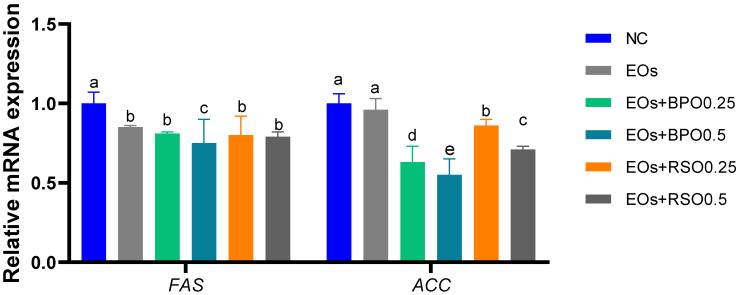
Effect of EOs with black pepper oil or radish seeds oil on mRNA expression of fatty acid synthase (*FAS*) and acetyl–coA carboxylase (ACC); ^abcde^ means within the same column carrying different superscripts are significantly different at (*p* < 0.05). EOs mixture of mint, parsley, and carrot oils. NC: feed basal diet without additives; EOs: birds fed a basal diet supplemented with 1.5 g/kg EOs; EOs + BPO0.25: birds fed a basal diet supplemented with 1.5 g/kg EOs plus 0.25 g/kg black pepper oil (BPO); EOs+BPO0.5: birds fed a basal diet supplemented with 1.5 g/kg EOs plus 0.25 g/kg black pepper oil (BPO); EOs + RSO0.25: birds fed a basal diet supplemented with 1.5 g/kg EOs plus 0.25 g/kg radish seed oil (RSO); EOs + RSO0.5: birds fed a basal diet supplemented with 1.5 g/kg EOs plus 0.5 g/kg radish seed oil (RSO).

Data related to the mRNA expression of the *FAS* and *ACC* genes are shown in Figure 2. The results reveal that the mRNA expression of the *FAS* gene was reduced (*p* < 0.05) in the groups supplemented with the EOs plus 0.5 g/kg of BPO (reduced by 0.75 fold). In addition, the broiler chickens that were fed EOs plus BPO at both levels had the lowest significant expression of the *ACC* gene (reduced by 0.63 and 0.55 fold).

### 3.7. Expression of Immune- and Autophagy-Related Genes

**Figure 3:**
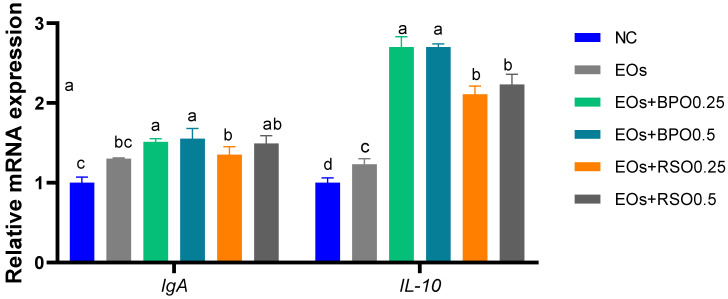
Effect of EOs with black pepper oil or radish seeds oil on mRNA expression of immunoglobulin A (IgA) and interleukin-10 (IL-10) genes; ^abcd^ means within the same column carrying different superscripts are significantly different at (*p* < 0.05). EOs mixture of mint, parsley, and carrot oils. NC: feed basal diet without additives; EOs: birds fed a basal diet supplemented with 1.5 g/kg EOs; EOs + BPO0.25: birds fed a basal diet supplemented with 1.5 g/kg EOs plus 0.25 g/kg black pepper oil (BPO); EOs + BPO0.5: birds fed a basal diet supplemented with 1.5 g/kg EOs plus 0.25 g/kg black pepper oil (BPO); EOs + RSO0.25: birds fed a basal diet supplemented with 1.5 g/kg EOs plus 0.25 g/kg radish seed oil (RSO); EOs + RSO0.5: birds fed a basal diet supplemented with 1.5 g/kg EOs plus 0.5 g/kg radish seed oil (RSO).

The expression patterns of IL-10 and IgA genes are described in Figure 3. The results revealed that all groups supplemented with EOs exhibited higher mRNA expression levels of IL-10 and IgA genes (*p* < 0.05) when compared with the NC group. Additionally, the group supplemented with EOs plus BPO (at both levels), followed by 0.5 g/kg of RSO, showed a significant increase in (*p* < 0.05) mRNA expression of IL-10 and IgA genes.

**Figure 4:**
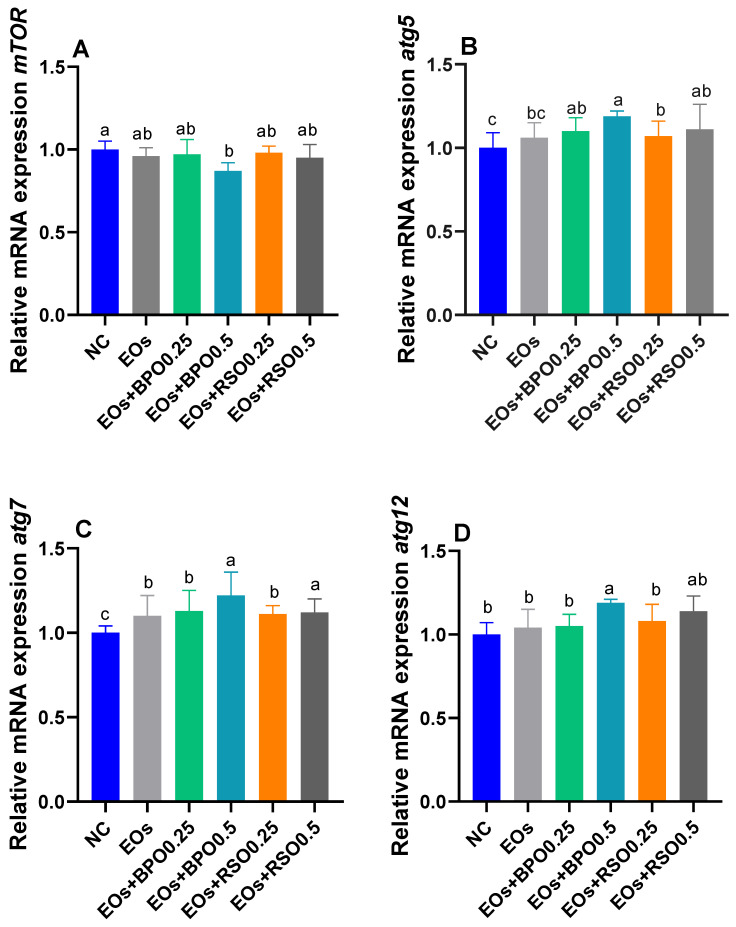
Effect of EOs with black pepper oil or radish seeds oil on mRNA expression of mechanistic target of rapamycin (mTOR, (**A**)) and autophagy-related genes (*atg5*, *atg7*, and *atg12* shown in (**B**–**D**), respectively) genes; ^abcd^ means within the same column carrying different superscripts are significantly different at (*p* < 0.05). EOs mixture of mint, parsley, and carrot oils. NC: feed basal diet without additives; EOs: birds fed a basal diet supplemented with 1.5 g/kg EOs; EOs + BPO0.25: birds fed a basal diet supplemented with 1.5 g/kg EOs plus 0.25 g/kg black pepper oil (BPO); EOs + BPO0.5: birds fed a basal diet supplemented with 1.5 g/kg EOs plus 0.25 g/kg black pepper oil (BPO); EOs + RSO0.25: birds fed a basal diet supplemented with 1.5 g/kg EOs plus 0.25 g/kg radish seed oil (RSO); EOs + RSO0.5: birds fed a basal diet supplemented with 1.5 g/kg EOs plus 0.5 g/kg radish seed oil (RSO).

The mRNA expression levels of mTOR and autophagy-related genes (*atg5*, *atg7*, and *atg12*) are illustrated in Figure 4A–D, respectively. The group that received the EOs plus 0.5 g/kg of BPO, followed by the group that received the EOs plus 0.5 g/kg of RSO, displayed patterns of higher expression for *atg5*, *atg7*, and *atg12*. By contrast, these groups showed the lowest expression of mTOR. 

## 4. Discussion

Recently, it was proved that phytobiotics based on EOs could be a practical alternative to antibiotics [42,43]. In poultry farming, the potential of the therapeutic application of EOs is attributable to their growth-promoting and immunomodulatory properties and antimicrobial and hypocholesterolemic activities [44,45]. Additionally, they can enhance digestive secretions, increase nutrient uptake, stimulate blood circulation, and have antioxidant effects [46]. A few studies had investigated the role of dietary individual supplementation with peppermint, parsley, carrot, black pepper, and radish seed EOs in broiler chickens [16,24]. However, the effects of their combination on broiler chickens, especially at the molecular level, had not been investigated until now. In the current study, supplementation with EO blends improved the cumulative BWG and feed efficiency of birds. Furthermore, the most prominent effect was detected in the group fed the EOs plus BPO or RSO at the level of 0.5 g/kg. Additionally, the latter groups exhibited the most improved FCR and nutrient digestibility, proving that such a combination of EOs had an imperative effect on the conversion of digested feed into body gain. In the same line, the pancreatic expression of *AMY2A*, *PNLIP*, and *CELA1* and duodenal expression of *CCK* were upregulated after feeding with the EO combination, especially at higher levels. Moreover, the addition of 0.5 g/kg of BPO to the EO blend had a prominent effect in upregulating *PNLIP* and *CCK* expression, compared with that observed in the other EO-supplemented groups. Accordingly, previous studies revealed that adding EOs to broiler chickens’ diets improved their performance parameters, as evinced by enhanced BWG, digestibility, and immune status [47,48,49,50,51,52]. It has been described that the dietary feeding of EOs extracted from herbs stimulated the secretion of pancreatic digestive enzymes in broiler chickens [53]. Other studies with chickens showed that a mixture of commercial EO components boosted the secretion and activities of digestive enzymes and, consequently, nutrient digestibility [49,54,55,56,57], which accord with our results for nutrient digestibility and the expression of digestive-enzyme-related genes. Additionally, an essential oil combination can increase trypsin, chymotrypsin, and elastase activities in broiler chickens [58,59].

Furthermore, peppermint leaves significantly enhanced feed intake and FCR [60]. This is probably due to their high content of menthol, the main active compound that increases appetite and feed efficiency in broiler chicks. Other active components present in peppermints such as citral, cineole, geraniol, and linalool have been found to have stimulatory effects on both nutrient digestion and absorption [31,60,61]. Additionally, supplementation with cold-pressed carrot seed oil caused a higher weight gain and carcass yield for broiler chickens, owing to its active ingredients, consisting of steroids, tannins, flavonoids, carotene, carotol, and limonene [62], with a lipotropic effect. On the other hand, the current data show that the addition of BPO at 0.5 g/kg had a positive effect on the growth performance of broiler chickens. The results were corroborated by [63], reporting that black pepper enhanced the performance, feed conversion ratio, and carcass-meat safety and quality in animals. Black pepper improved nutrient digestibility by increasing digestive liquids in the stomach and decreasing pathogenic microorganisms in broiler chickens [17]. Correspondingly, [64] described that ground black pepper with different levels led to improved digestibility for dry matter, crude protein, and ether extract in broiler chickens. Additionally, the principal active compound in black pepper, piperine, stimulated pancreatic digestive enzymes that had an important role in improving digestion and feed conversion [4]. Additionally, feeding broiler chickens with an EO blend consisting of thymol, eugenol, and piperine boosted the total activity of amylase, maltase, and trypsin, unlike the control treatment [53]. Feeding with a plant extract comprising capsaicin, cinnamaldehyde, and carvacrol stimulated the absorption of amino acids [65]. This can be explained by their antioxidative activity, which protects microvilli, responsible for nutrient absorption [66,67]. Remarkably, the higher growth rate in the group fed with the EOs plus RSO at 0.5 g/kg may be related to the high contents of flavonoids and anthocyanin, the most important components of radish seed oil [18], which protect living cells from oxidative stress, consequently resulting in the good productive performance of broiler chickens. The present results are consistent with [68], who reported that the addition of radish roots to the layer diet improved the layers’ performance by increasing the egg number and egg mass. The data reported in the research of [20] show that radish root extract contains the peroxidase enzyme and anthocyanins, which have a radical-scavenging activity that may increase nutrient utilization and increase the growth rate of broiler chickens. From the present results, it could be postulated that supplementation with EOs at higher levels could trigger the secretion of pancreatic enzymes under certain conditions, which could result in better nutrient digestion in the intestine.

The intestinal microflora has an important function of protecting intestinal mucosal integrity [69]. Antibacterial activity against less-favorable and pathogenic bacteria residing in the gut is considered one of the main essential biological functions of phytogenic components [70]. Herein, our results show that supplementation with an EO blend with either BPO or RSO at higher levels had favorable effects on the gut microflora by enhancing the counts of beneficial lactobacilli and lowering the population of *coliform*. Accordingly, a blend of EOs (thyme, peppermint, savory, and black pepper) altered the gut ecosystem to favor beneficial microflora [71]. Similarly, phytogenic products such as EOs could act against the colonization of intestinal pathogens such as *Clostridium perfringens* and *E. coli* due to their antibacterial effects in poultry [72,73]. Moreover, supplementation with carrot seed oil increased the number of lactic acid bacteria but did not change the number of *E. coli* bacteria [62]. A mixture of cinnamaldehyde, carvacrol, and capsicum oleoresin increased the lactobacilli population and cecal jejunal ratio of lactobacilli to Enterobacteria [74,75] of early weaned piglets. Likewise, the antibacterial properties of EOs against pathogens such as *Salmonella typhi*, *E. coli*, and *Staphylococcus aureus* has been demonstrated in vitro [76,77]. Additionally, a combination of EOs can enhance beneficial bacterial proliferation, as evinced by increased lactic acid bacterial counts in the gut digesta [78]. The general suggested mechanisms of action of EOs are disrupting bacterial-cell permeability [79], changing the pH in the bacterial cell [80], and supplying the substrates for the growth and proliferation of intestinal lactic acid bacteria such as *Lactobacillus* [81]. Additionally, the antimicrobial action of EOs is mediated by their lipophilic properties, which enable them to perforate the bacterial-cell membrane, causing the release of the bacterium’s components to the external environment [82]. Another proven mode of action of EOs is stimulating the production of intestinal mucus in broiler chickens, which hinders the adhesion of pathogenic bacteria and contributes to stabilizing gut microbial eubiosis in poultry [79].

The serum total protein, albumin, and globulin levels were enhanced by supplementation with the EO blend, especially with higher levels of BPO or RSO. Consistent with our results, [47] reported that black pepper supplementation improved the serum total protein and albumin in broiler chickens. The serum concentrations of AST and ALT indirectly reflect the liver’s health status, and increases in their concentrations are considered indicators of liver damage. Herein, the serum levels of AST and ALT were not affected by dietary supplementation with the EO blend and were within the normal ranges, which indicates healthy liver functions in both the control and EO-supplemented groups. In agreement with the data of [47], black pepper and the other herbs did not affect liver enzymes. Interestingly, the concentrations of TAG, total cholesterol, VLDL cholesterol, and LDL cholesterol were prominently decreased, while HDL cholesterol was increased, in the groups supplemented with the EOs with BPO. In the same line, the decrease in the serum levels of cholesterol and triglycerides was accompanied by the downregulation of *FAS* and *ACC* expression, especially in groups supplemented with the EOs with BPO or RSO. A possible mechanism for the reduction in the synthesis of cholesterol by phytogenic EOs is the inhibitory effects of their active agents on the reductase enzyme 3-hydroxy-3-methylglutaryl-CoA or enhanced lipoprotein catabolism [72]. Additionally, the current results are supported by [83], who reported that the addition of protein isolates from radish leaves decreased the cholesterol and triglyceride levels in broiler serum; this effect may be attributed to the greater fecal excretion by these animals, inhibiting the intestinal absorption of cholesterol and bile. Additionally, feeding with EOs plus 0.5 g/kg of BPO markedly reduced the expression levels of *FAS* and *ACC* (decreased by 0.75 and 0.55 fold vs. the control group). These findings indicate the lipotropic effect of BPO, evinced by the changes in *FAS* and *ACC* expression in adipose tissues. A previous study clearly demonstrated the role of black pepper in lipid metabolism [84]. Furthermore, [16] reported that black pepper powder lowered TC and TAG in the serum, and this may be attributed to the inhibition of acetyl–CoA synthesis, which is necessary for triglyceride synthesis. Additionally, [85] indicated that a fat-soluble extract of Japanese radish increased the levels of HDL cholesterol, which has an antiarteriosclerotic effect. 

The immune system plays an important role in maintaining broiler chickens’ health. Essential oils positively affect the avian immune system, since they stimulate immunoglobulin production, enhance the activity of lymphocytes, and boost the release of interferon-γ [86,87]. In this context, the addition of an EO blend to broiler chickens’ feed improved serum immune parameters, as evinced by increased lysozyme activity, immunoglobulin levels, and phagocytic indices in comparison with the NC group; moreover, their highest levels were detected for BPO-supplemented groups over a 35-day rearing period. Similarly, [88] showed that EOs such as mint oils have potent immunomodulatory effects in chickens. In addition, supplementing with EOs enhanced the proliferation rate for serum lymphocytes, phagocytosis, and IgG, IgM, and IgA levels in piglets [89]. Herein, the expression levels of the IL-10 and IgA genes were upregulated simultaneously with enhanced immune serum parameters after feeding with the EO blend with BPO. Accordingly, the piperine contained in BPO directly activates the immune system [11,90]. From our point of view, piperine has antimicrobial, anti-inflammatory, and antioxidant activities that improve serum humoral and cellular immunity, with the upregulation of the expression of IL-10 and IgA, consequently decreasing the proliferation of pathogenic bacteria and inflammation in broiler chickens, in accordance with [47]. On the other hand, autophagy is a critical pathway that sustains cellular homeostasis and physiological processes, including reproduction, development [91,92], and immunity [93]. Additionally, it assists as a cellular defense mechanism against external harmful stimuli via the degradation of protein aggregates, damaged organelles, and even pathogens in cells [94]. The malfunction of autophagy is related to diverse diseases such as neurodegeneration [95], metabolic syndrome, and inflammation [92]. The initiation of autophagy depends on the participation of a series of autophagy-related genes *(atg*), such as mTOR, microtubule-associated protein 1 light chain 3 (LC3), *atg5*, *atg7*, and *atg12*. The mTOR gene promotes protein synthesis and impedes autophagy initiation, whereas the elevation of AMPKα1 accelerates energy production through autophagy, glycolysis, and lipolysis, inhibiting energy-exhausting pathways (e.g., protein synthesis) [96]. The expression of autophagy-related genes was markedly upregulated in the BPO-supplemented groups. Additionally, in the current study, it was detected that the downregulation of mTOR was inhibited by the addition of EOs. In the same line, the BPO-supplemented groups showed the most prominent downregulation of mTOR expression (decreased by 0.87 fold). These findings prove the beneficial role of the EO blend with BPO in modulating the mechanisms of autophagy inside the bird’s body. Accordingly, piperine enhanced autophagy by inhibiting mTOR via protein phosphatase 2A activation [97]. In this context, phagocytosis was significantly increased upon the addition of BPO to EOs. Accordingly, dietary supplementation with essential oils increased phagocytic activity and phagocytic index in broiler chickens [98]. 

## 5. Conclusions

We found that groups fed EOs + BPO or EOs + RSO at 0.5 g/kg diet showed the maximum growth-promoting effects in the current study, which were realized by its stimulating effect on both the expression of digestive-enzyme-related genes and nutrient digestion. Moreover, supplementation at 0.5 g/kg diet with EOs + BPO upregulated the expression of the *PNLIP* gene and markedly regulated lipogenesis, as proved by the downregulation of the *FAS* and *ACC* genes. The immune-boosting effect among experimental groups was more obvious in the groups supplemented with EOs + BPO than EOs + RSO. Additionally, dietary EOs + BPO supplementation stimulated the expression of autophagy-related genes and increased the phagocytic %, indicating that it could influence cellular immunity. Finally, using a combination of EOs plus 0.5 g/kg diet of EOs + BPO is recommended for broiler chickens’ diets due to its digestive and immune-stimulating effects.

## Figures and Tables

**Table 1:** Composition of experimental diet.

	Experimental Diets		
	Starter	Grower	Finisher
Yellow corn	57.40	60.10	62.00
Soybean meal, 47.5%	34.66	29.00	25.00
Corn gluten, 59.3%	3.00	4.00	4.00
Wheat bran	-	-	1.90
Soybean oil	1.10	3.00	3.66
Calcium carbonate	1.00	1.00	0.90
Dicalcium phosphate	1.80	1.90	1.60
Common salt	0.30	0.30	0.30
Premix ^1^	0.30	0.30	0.30
DL-Methionine, 98%	0.18	0.14	0.11
Lysine, HCL, 78%	0.16	0.16	0.13
Antimycotoxin	0.10	0.10	0.10
Calculated composition			
ME, Kcal/Kg	3004.02	3157.17	3202.02
CP, %	23.01	21.10	19.57
EE, %	3.63	5.55	6.24
CF, %	2.66	2.53	2.64
calcium, %	0.97	0.98	0.86
Available P%	0.47	0.47	0.41
Lysine, %	1.37	1.22	1.10
Methionine, %	0.56	0.51	0.46

^1^ Muvco premix: Each 2.5 kg contain vit. A (10, 000000 IU), vit. D3 (2, 000000 IU), vit. E (10 g), vit. k3 (1000 mg), vit. B1 (1000 mg), vit. B2 (5 g), vit. B6 (1.5 g), pantothenic acid(10 g),vit. B12 (10 mg), niacin (30 g), folic acid (1000 mg), biotin (50 mg), fe (30 g), Mn (60 g), Cu (4 g), I (300 mg), Co (100 mg), Se (100 mg), and Zn (50 g). ME, metabolic energy; CP: crude protein; EE: ether extract; CF: crude fiber.

**Table 2:** Primer sequences used for gene expression analysis by RT–qPCR assay.

Gene	Primer Sequence (5′-3′)	Accession No.
Digestive-enzyme-related genes		
*AMY2A*	F: CGGAGTG↓GATGTTAACGACTGG R: ATGTTCGCAGACCCAGTCATTG	NM_001001473.2
*PNLIP*	F: GCATCTGGGAAG↓GAACTAGGG R. TGAACCACAAGCATAGCCCA	NM_001277382.1
*CELA1*	F: AGCGTAAGGAAATGGGGTGG R. GTGGAGACCCCATGCAAGTC	XM_017007509.2
*CCK*	F: AGGTTCCACTGGGAGGTTCT R: CGCCTGCTGTTCTTTAGGAG	XM_015281332.1
Lipogenesis genes		
*FAS*	F: GCAGCTTCGGTGCCTGTGGTT R: GCTGCTTGGCCCACACCTCC	NM205155
*ACC*	F: TGCCTCCGAGAACCCTAA R: TCCAGGCTTGATACCACA	JQ080306
Immune-related genes		
*IL-10*	F: GCTGAGGGTGAAGTTTGAGG R: AGACTGGCAGCCAAAGGTC	XM_025143715.1
*IgA*	F: ACCACGGCTCTGACTGTACC R: CGATGGTCTCCTTCACATCA	S40610.1
Autophagy-related genes		
mTOR	F: CATGTCAGGCACTGTGTCTATTCTC R: CTTTCGCCCTTGTTTCTTCACT	XM_417614.5
*Atg5*	F: TCACCCCTGAAGATGGAGAGA R: TTTCCAGCATTGGCTCAATTC	NM_001006409
Atg 7	F: ACTGGCAATGCGTGTTTCAG R: CGATGAACCCAAAAGGTCAGA	NM_001030592
*Atg12*	F: GCACCCGCACCATCCA R: GAGGCCATCAGCTTCAGGAA	XM_003643073
Housekeeping		
*GAPDH*	F: CAACCCCCAATGTCTCTGTT R: TCAGCAGCAGCCTTCACTAC	NM205518

AMY2A: pancreatic alpha 2A amylase; CELA1: chymotrypsin-like elastase family, member 1; *CCK*: cholecystokinin; *FAS*: fatty acid synthase gene; *ACC*: acetyl–coA carboxylase; *IL-10*: interleukin-10; *IgA*: immunoglobulin A; mTOR: mechanistic target of rapamycin; Atg: autophagy-related genes; *GAPDH*: glyceraldehyde-3-phosphate dehydrogenase.

**Table 3:** Effect of EOs with black pepper oil or radish seeds oil on growth performance of broiler chickens.

Parameters	Experimental Groups						SEM	*p*-Value
	NC	EOs	EOs + BPO0.25	EOs + BPO0.5	EOs + RSO0.25	EOs + RSO0.5		
Initial BW (g/bird)	44.40	44.5	44.94	44.96	44.54	44.76	0.16	0. 904
Final BW (g/bird)	2259 ^d^	2375 ^c^	2378 ^c^	2422 ^ab^	2411 ^b^	2466 ^a^	21.11	<0.001
BWG (g/bird)	2215 ^d^	2331 ^c^	2333 ^c^	2377 ^ab^	2366 ^b^	2421 ^a^	21.14	<0.001
FI (g/bird)	3899	3893	3830	3825	3843	3879	9.71	0.075
FCR	1.76 ^a^	1.67 ^b^	1.64 ^bc^	1.61 ^c^	1.62 ^c^	1.60 ^c^	0.02	0.014
RGR (%)	192.29	192.63	192.58	192.69	192.74	192.85	0.07	0.08
PER	2.80 ^c^	2.94 ^b^	3.00 ^ab^	3.07 ^a^	3.04 ^a^	3.08 ^a^	0.03	<0.001

BW: body weight; BWG: body weight gain; FI: feed intake; FCR: feed conversion ratio; RGR: relative growth rate; PER: protein efficiency ratio. NC (negative control): birds fed a basal diet without additives. EOs mixture of mint, parsley, and carrot seed oils. EOs: birds fed a basal diet supplemented with 1.5 g/kg EOs; EOs + BPO0.25: birds fed a basal diet supplemented with 1.5 g/kg EOs plus 0.25 g/kg black pepper oil (BPO); EOs + BPO0.5: birds fed a basal diet supplemented with 1.5 g/kg EOs plus 0.25 g/kg black pepper oil (BPO); EOs + RSO0.25: birds fed a basal diet supplemented with 1.5 g/kg EOs plus 0.25 g/kg radish seed oil (RSO); EOs + RSO0.5: birds fed a basal diet supplemented with 1.5 g/kg EOs plus 0.5 g/kg radish seed oil (RSO). SEM: standard error of the mean; ^abcd^: means with different superscripts within the same row differ significantly (*p* < 0.05).

**Table 4:** Effect of EOs with black pepper oil or radish seeds oil on nutrient digestibility.

	Experimental Groups							
Digestion Coefficient, %	NC	EOs	EOs + BPO0.25	EOs + BPO0.5	EOs + RSO0.25	EOs + RSO0.5	SEM	*p*-Value
DM	77.64 ^b^	79.00 ^b^	82.07 ^a^	82.99 ^a^	81.04 ^a^	81.68 ^a^	0.49	<0.001
CP	70.75 ^d^	71.43 ^c^	72.780 ^b^	74.19 ^a^	72.37 ^b^	73.51 ^ab^	0.36	0.029
EE	80.36 ^e^	81.87 ^d^	83.20 ^b^	85.74 ^a^	81.61 ^cd^	82.74 ^c^	0.45	<0.001

DM: dry matter; CP: crude protein; EE: ether extract. EOs mixture of mint, parsley, and carrot oils. EOs: birds fed a basal diet supplemented with 1.5 g/kg EOs; EOs + BPO0.25: birds fed a basal diet supplemented with 1.5 g/kg EOs plus 0.25 g/kg black pepper oil (BPO); EOs+BPO0.5: birds fed a basal diet supplemented with 1.5 g/kg EOs plus 0.25 g/kg black pepper oil (BPO); EOs + RSO0.25: birds fed a basal diet supplemented with 1.5 g/kg EOs plus 0.25 g/kg radish seed oil (RSO); EOs + RSO0.5: birds fed a basal diet supplemented with 1.5 g/kg EOs plus 0.5 g/kg radish seed oil (RSO). SEM: standard error of the mean; ^abcd^: means with different superscripts within the same row differ significantly (*p* < 0.05).

**Table 5:** Effect of EOs with black pepper oil or radish seeds oil on serum biochemicals in broiler chickens.

	Experimental Groups							
Parameters	NC	EOs	EOs + BPO0.25	EOs + BPO0.5	EOs + RSO0.25	EOs + RSO0.5	SEM	*p*-Value
Total protein (g/ dL)	3.32 ^d^	3.38 ^c^	3.50 ^b^	3.77 ^a^	3.49 ^b^	3.80 ^a^	0.03	<0.001
Albumin (g/dL)	1.75 ^e^	1.80 ^d^	1.87 ^c^	1.93 ^b^	1.88 ^c^	1.97 ^a^	0.01	<0.001
Globulin (g/ dL)	1.58 ^c^	1.58 ^bc^	1.63 ^b^	1.83 ^a^	1.61 ^bc^	1.83 ^a^	0.02	<0.001
Albumin/globulin ratio	1.11 ^bc^	1.14 ^ab^	1.15 ^a^	1.06 ^d^	1.16 ^a^	1.07 ^cd^	0.01	<0.001
TAG (mg/ dL)	60.68 ^a^	57.08 ^b^	54.54 ^d^	54.09 ^d^	55.33 ^c^	54.87 ^cd^	0.42	<0.001
Total cholesterol (mg/ dL)	131.00 ^a^	124.45 ^b^	119.74 ^d^	119.39 ^d^	122.12 ^c^	121.73 ^c^	0.74	<0.001
HDL (mg/ dL)	90.17 ^e^	96.03 ^d^	102.92 ^b^	105.59 ^a^	98.80 ^c^	99.88 ^c^	0.93	<0.001
VLDL (mg/ dL)	12.14 ^a^	11.42 ^b^	10.91 ^d^	10.82 ^d^	11.07 ^c^	10.97 ^cd^	0.08	<0.001
LDL (mg/ dL)	28.69 ^a^	17.00 ^b^	2.99 ^f^	5.92 ^e^	12.26 ^c^	10.87 ^d^	1.56	<0.001
ALT (U/L)	18.00	17.92	18.22	19.12	18.06	18.78	0.19	0.40
AST (U/L)	17.76	18.42	17.92	18.94	18.48	18.82	0.19	0.41

TAG: triacylglycerol; HDL: high-density lipoprotein; VLDL: very-low-density lipoprotein; LDL: low-density lipoprotein; ALT: alanine aminotransferase; AST: aspartate aminotransferase. EOs mixture of mint, parsley, and carrot seed oils. EOs: birds fed a basal diet supplemented with 1.5 g/kg EOs; EOs + BPO0.25: birds fed a basal diet supplemented with 1.5 g/kg EOs plus 0.25 g/kg black pepper oil (BPO); EOs + BPO0.5: birds fed a basal diet supplemented with 1.5 g/kg EOs plus 0.25 g/kg black pepper oil (BPO); EOs + RSO0.25: birds fed a basal diet supplemented with 1.5 g/kg EOs plus 0.25 g/kg radish seed oil (RSO); EOs + RSO0.5: birds fed a basal diet supplemented with 1.5 g/kg EOs plus 0.5 g/kg radish seed oil (RSO). SEM: standard error of the mean; ^abcdef^ means with different superscripts within the same row differ significantly (*p* < 0.05).

**Table 6:** Effect of EOs with black pepper oil or radish seeds oil on serum immune parameters in broiler chickens.

	Experimental Groups							
Parameters	NC	EOs	EOs + BPO0.25	EOs + BPO0.5	EOs + RSO0.25	EOs + RSO0.5	SEM	*p*-Value
Lysozymes (μ/mL)	0.79 ^d^	0.81 ^d^	0.86 ^b^	1.00 ^a^	0.83 ^c^	0.87 ^b^	0.01	<0.001
IgM (mg/dL)	13.26 ^d^	14.16 ^c^	16.11 ^b^	18.10 ^a^	14.44 ^c^	16.27 ^b^	0.31	<0.001
IgG (mg/dL)	1.68 ^d^	1.84 ^c^	2.04 ^b^	2.40 ^a^	1.84 ^c^	2.06 ^b^	0.05	<0.001
Phagocytic, %	61.28 ^d^	63.86 ^c^	68.56 ^b^	83.46 ^a^	64.68 ^c^	68.88 ^b^	1.37	<0.001

IgM: immunoglobulins M; IgG: immunoglobulins G. EOs mixture of mint, parsley, and carrot oils. EOs: birds fed a basal diet supplemented with 1.5 g/kg EOs; EOs + BPO0.25: birds fed a basal diet supplemented with 1.5 g/kg EOs plus 0.25 g/kg black pepper oil (BPO); EOs + BPO0.5: birds fed a basal diet supplemented with 1.5 g/kg EOs plus 0.25 g/kg black pepper oil (BPO); EOs + RSO0.25: birds fed a basal diet supplemented with 1.5 g/kg EOs plus 0.25 g/kg radish seed oil (RSO); EOs + RSO0.5: birds fed a basal diet supplemented with 1.5 g/kg EOs plus 0.5 g/kg radish seed oil (RSO). SEM: standard error of the mean; ^abcd^ means with different superscripts within the same row differ significantly (*p* < 0.05).

**Table 7:** Effect of EOs with black pepper oil or radish seeds oil on cecal bacterial count in broiler chickens (Log CFU/g).

Parameters	Experimental Groups							
	NC	EOs	EOs + BPO0.25	EOs + BPO0.5	EOs + RSO0.25	EOs + RSO0.5	SEM	*p*-Value
Total bacterial count	15.39 ^a^	13.51 ^b^	13.50 ^b^	11.41 ^c^	13.65 ^b^	12.00 ^c^	0.32	<0.001
Coliforms count	7.39 ^a^	5.94 ^b^	5.50 ^c^	4.35 ^e^	5.66 ^bc^	5.00 ^d^	0.23	<0.001
Lactobacilli count	5.36 ^e^	7.28 ^d^	7.91 ^cd^	9.69 ^a^	8.05 ^c^	8.79 ^b^	0.33	<0.001

EOs mixture of mint, parsley, and carrot oils. EOs: birds fed a basal diet supplemented with 1.5 g/kg EOs; EOs + BPO0.25: birds fed a basal diet supplemented with 1.5 g/kg EOs plus 0.25 g/kg black pepper oil (BPO); EOs + BPO0.5: birds fed a basal diet supplemented with 1.5 g/kg EOs plus 0.25 g/kg black pepper oil (BPO); EOs + RSO0.25: birds fed a basal diet supplemented with 1.5 g/kg EOs plus 0.25 g/kg radish seed oil (RSO); EOs + RSO0.5: birds fed a basal diet supplemented with 1.5 g/kg EOs plus 0.5 g/kg radish seed oil (RSO). SEM: standard error of the mean; ^abcde^ means with different superscripts within the same row differ significantly (*p* < 0.05).

## Data Availability

The data presented in this study are available on request from the corresponding author.
